# Supplementary material for: Gene activation guided by nascent RNA-bound transcription factors
Source: Nat Commun. 2022 Nov 28;13:7329. doi: 10.1038/s41467-022-35041-7 (PMC9705438; doi:10.1038/s41467-022-35041-7)
Supplement: Supplementary file 1 — Supplemental information [file 41467_2022_35041_MOESM1_ESM.pdf]

Supplementary Materials for

Gene activation guided by nascent RNA-bound transcription factors

Ying Liang[1,2,†], Haiyue Xu[1,2,†], Tao Cheng[3], Yujuan Fu[1], Hanwei Huang[1], Wenchang Qian[4], Junyan Wang[1], Yuenan Zhou[5], Pengxu Qian[4], Yafei Yin[5], Pengfei Xu[3], Wei Zou[6,7,]* and Baohui Chen[1,2,8,9]*

*To whom correspondence should be addressed. Email: zouwei@zju.edu.cn; baohuichen@zju.edu.cn

The PDF file includes:

Fig. S1 Design of the Dox-inducible Narta activation system.

Fig. S2 Narta activates exogenous transgenes.

Fig. S3 Narta activates the exogenous reporter gene in Zebrafish embryos.

Fig. S4 Activation of endogenous genes by Narta in HeLa cells.

Fig. S5 Detection of Narta activation by smFISH.

Fig. S6 Activation of endogenous genes by Narta in 293T cells.

Fig. S7 Simultaneous activation of three endogenous genes by Narta.

Fig. S8 The optimal copy number of MS2 for Narta activation.

Fig. S9 Narta activation via intronic MS2 is more effective than UTR MS2.

Fig. S10 Effect of NarTag knockin on the protein expression of target genes.

Fig. S11 Selecting efficient sgRNAs to perform CRISPRa.

Fig. S12 Comparison and combinatory use of CRISPRa and Narta for gene activation.

Fig. S13 Comparison of Narta to other gene activation methods.

Fig. S14 Gating strategy of flow cytometry analysis

Table S1. CRISPR-Cas9 sgRNAs used in this study

Table S2. CRISPR-Cas13 crRNAs used in this study

Table S3. Primers used for qPCR

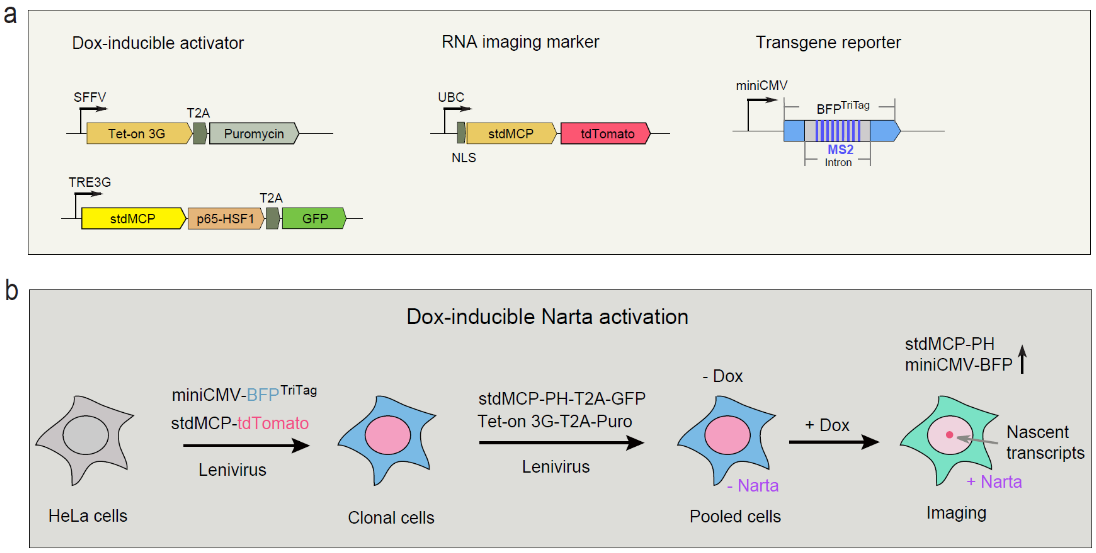

Supplementary Fig. 1 Design of the Dox-inducible Narta activation system. a, Schematic of construct designs to test the activation of a transgene reporter by Dox-inducible Narta. **b**, Work flow to generate a cell line for achieving Dox-inducible Narta activation. Firstly, HeLa cells were co-infected with the mixture of miniCMV-BFP^{TriTag} and stdMCP-tdTomato lentiviruses. A clonal cell line with optimal expression level of miniCMV-BFP^{TriTag} and stdMCP-tdTomato was then isolated. Secondly, this clonal cell line was infected with the mixed lentiviruses of stdMCP-PH-T2A-GFP and Tet-on 3G-T2A-Puromycin. Dox-inducible stdMCP-PH-T2A-GFP cells were isolated by Puromycin selection. Thus, stdMCP-PH-T2A-GFP can be expressed after the addition of Dox to induce Narta activation. Finally, the activation of transgene reporters can be monitored by fluorescent imaging of both nascent RNAs (labeled by stdMCP-tdTomato) and target protein expression levels (revealed by BFP).

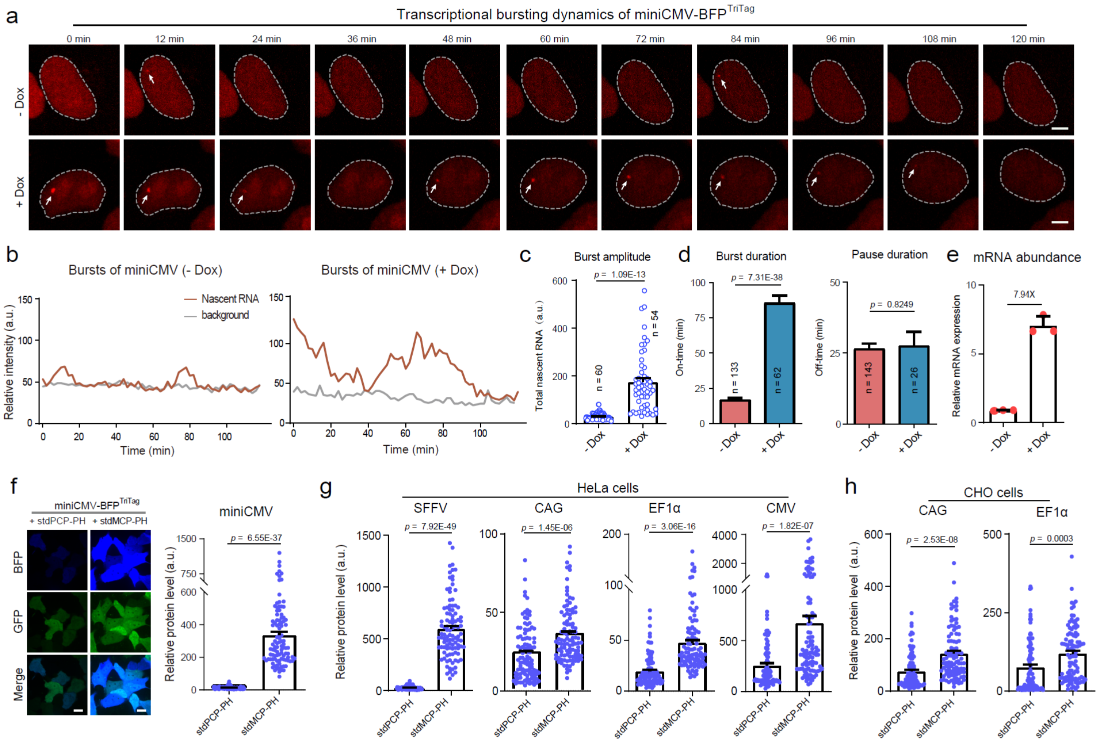

Supplementary Fig. 2 Narta activates exogenous transgenes. a, Snapshots of representative HeLa cells showing the transcriptional bursting dynamics of miniCMV-BFP^{TriTag} revealed by stdMCP-tdTomato without (top) or with (bottom) Narta activation. Fluorescent foci pointed by arrows represent nascent transcription sites. Scale bar, 5 µm. **b**, Fluorescent traces (red) of nascent transcripts produced at miniCMV loci from the cells in (B). Gray traces denote the background signal in the nuclei. **c**, Statistic of the burst amplitude of miniCMV defined by the maximum total intensity of individual stdMCP-tdTomato spots in each burst. **d**, Quantifications of burst durations (left) and pause durations (right) to show the bursting features of miniCMV transcription. In D and E, data are displayed as mean ± s.e.m. n = 100 cells. **e**, Measurement of mRNA abundance to reveal the transcriptional level of miniCMV by quantitative PCR. The error bars represent means ± s.e.m. of n = 3 biologically independent experiments. *P*-value was analyzed by two-tailed Student's *t*-test. **f**, Representative fluorescent images and quantifications to show BFP expression driven by miniCMV upon stdPCP-PH (negative control) or stdMCP-PH (inducing Narta activation) transfection. Scale bar, 10 µm. **g**,**h**, Quantifications of BFP expression driven by different promoters with or without Narta activation in HeLa (**g**) and CHO cells (**h**). Data were displayed as means ± s.e.m., n = 100 cells. *P*-value was analyzed by two-tailed Student's *t*-test. Source data are provided as a Source Data file.

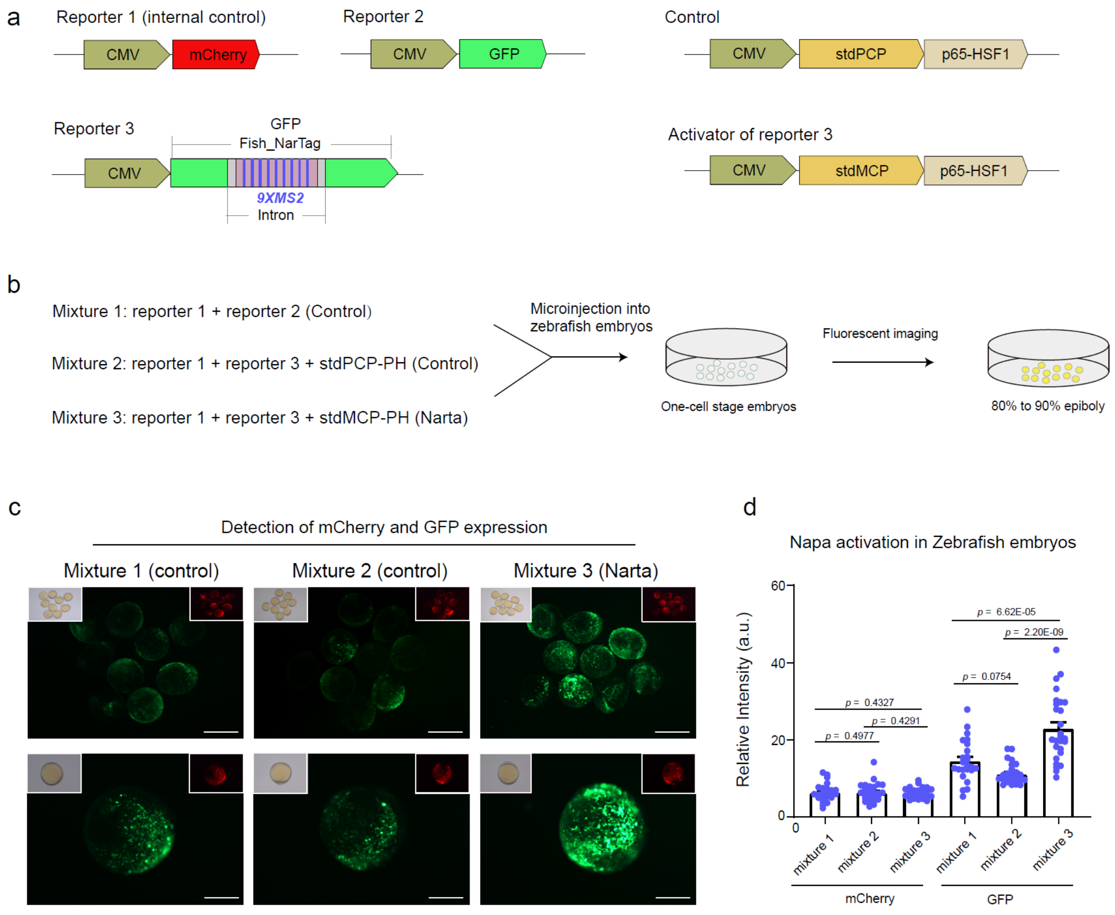

Supplementary Fig. 3 Narta activates the exogenous reporter gene in Zebrafish embryos. a, Schematic of construct designs to perform Narta in Zebrafish. **b**, Three plasmid mixtures as shown were prepared for the microinjection into zebrafish embryos. Imaging was performed about 10h after injection. **c**, Representative images showing the expression of CMV-mCherry, CMV-GFP and CMV-GFP$^{\text{Fish_NarTag}}$ without or with Narta activation. Scale bar of the large field images is 500 μm. The scale bar of zoomed-in view is 200 μm. **d**, Quantification of mCherry or GFP expression levels of the embryos from C. The constitutive mCherry expression was quantified as the internal control to assess the amount of DNA injected across the different conditions. Each dot represents a single embryo. For each group from left to right, n = 24, 28, 27, 24, 28 and 27 biologically independent embryos, respectively. Data show mean ± s.e.m. *P*-value was calculated by One-way ANOVA analysis (with Post Test, turkey). Source data are provided as a Source Data file.

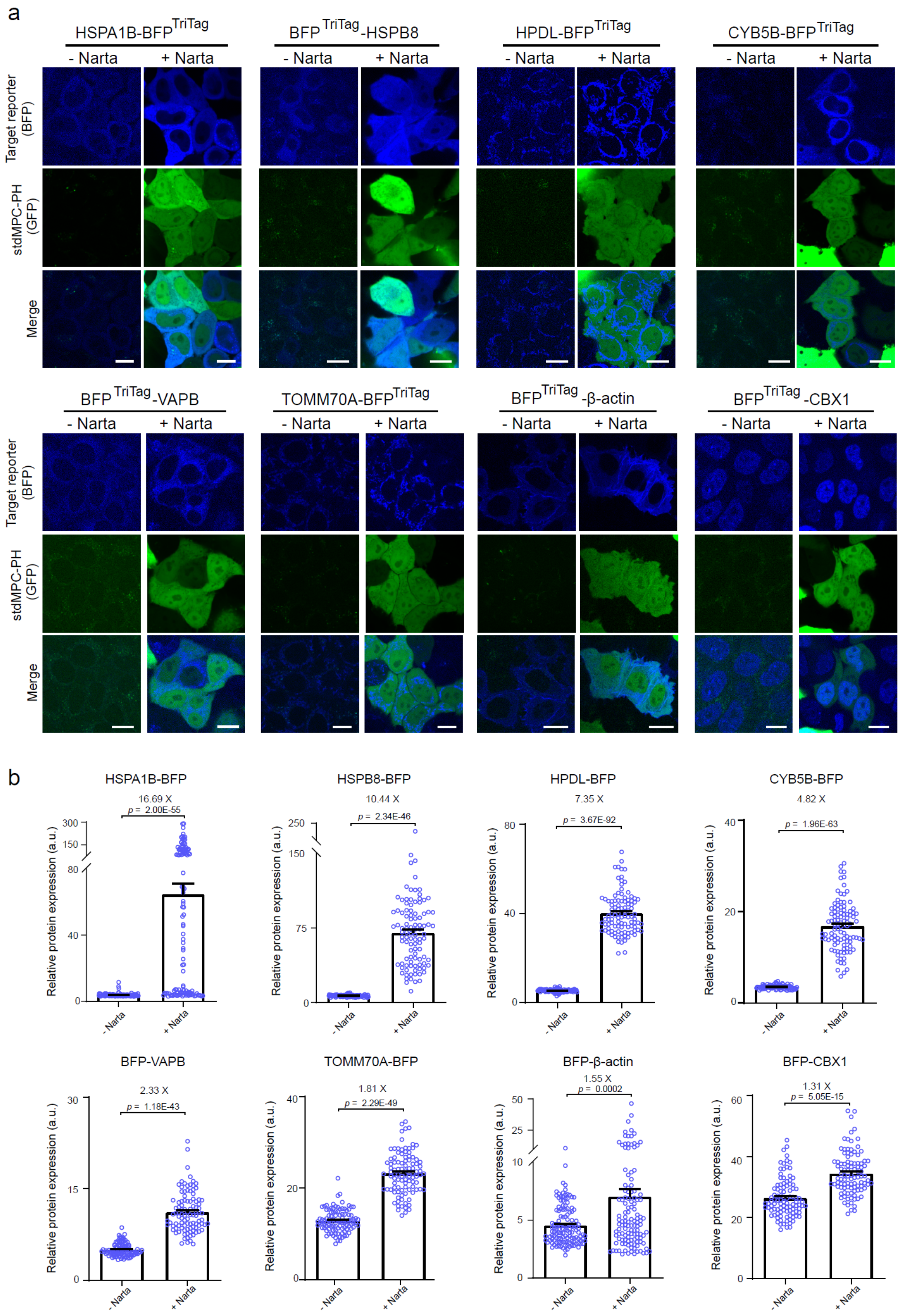

Supplementary Fig. 4 Activation of endogenous genes by Narta in HeLa cells. **a**, Representative images of endogenous reporter genes labeled by BFP^{TriTag} with or without Narta activation. StdMCP-PH-T2A-GFP was transfected into cells to induce Narta activation. Scale bar, 5 μm. **b**, Quantifications of protein expression levels based on fluorescent imaging in A. Each circle represents a single cell. n = 100 cells. All plots are shown as mean ± s.e.m. *P*-value was analyzed by two-tailed Student's *t*-test. Source data are provided as a Source Data file.

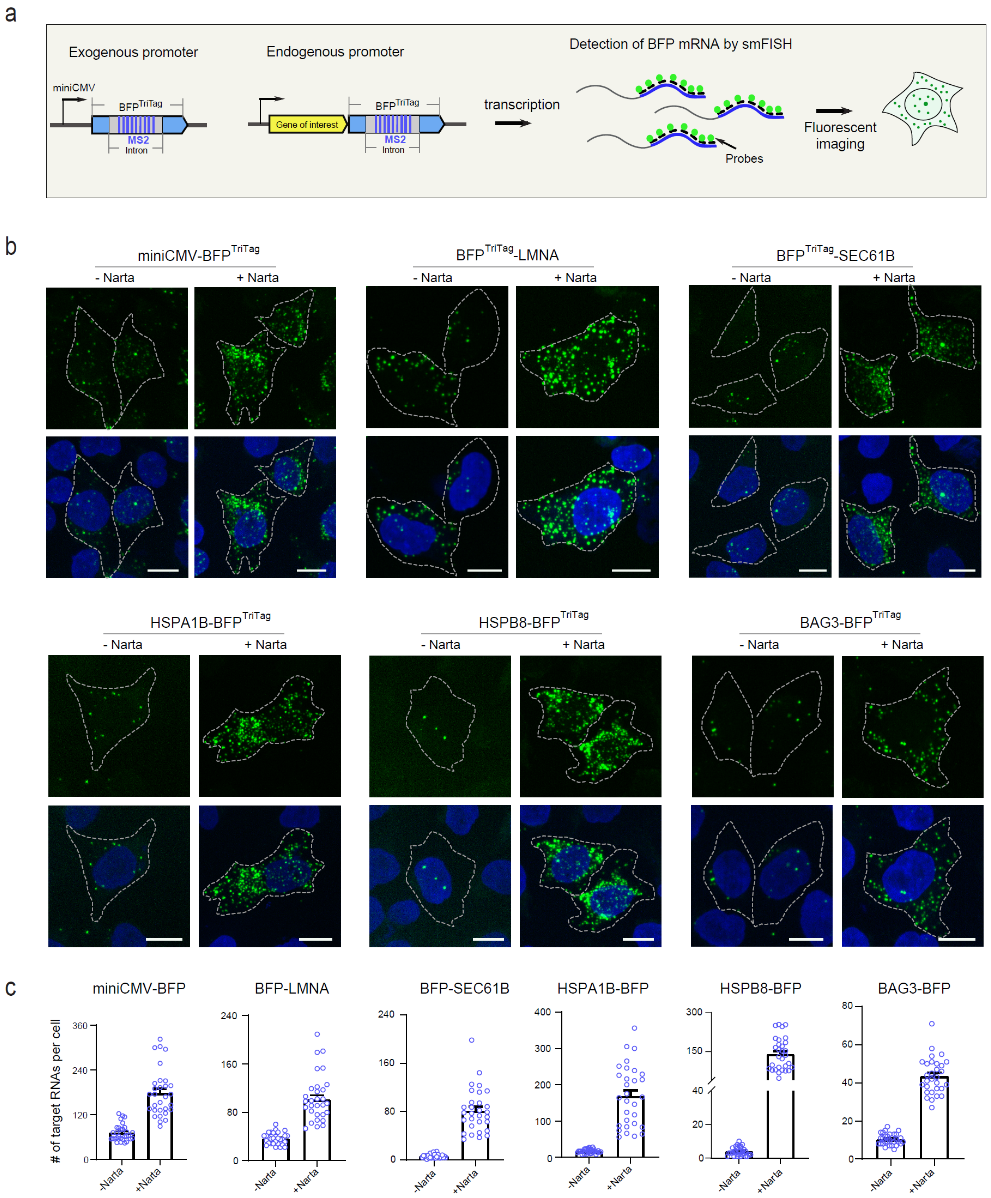

Supplementary Fig. 5 Detection of Narta activation by smFISH. a, Schematic illustration of the principle to detect BFP-fused mRNAs using single-molecule fluorescence in situ hybridization (smFISH). As indicated, FISH probes specifically recognize BFP sequence in the mRNA molecules. **b**, Representative fluorescent images to show the detection of BFP or BFP-fused mRNAs (green). Nuclei were stained by Hoechst 3332 (blue). Dotted lines outline the edge of cells. All images are maximum intensity projections from z stacks. Scale bar, 10 µm. **c**, Quantifications of mRNA number in each cell detected by smFISH in (b). n = 30 cells. All plots are shown as mean ± s.e.m. Source data are provided as a Source Data file.

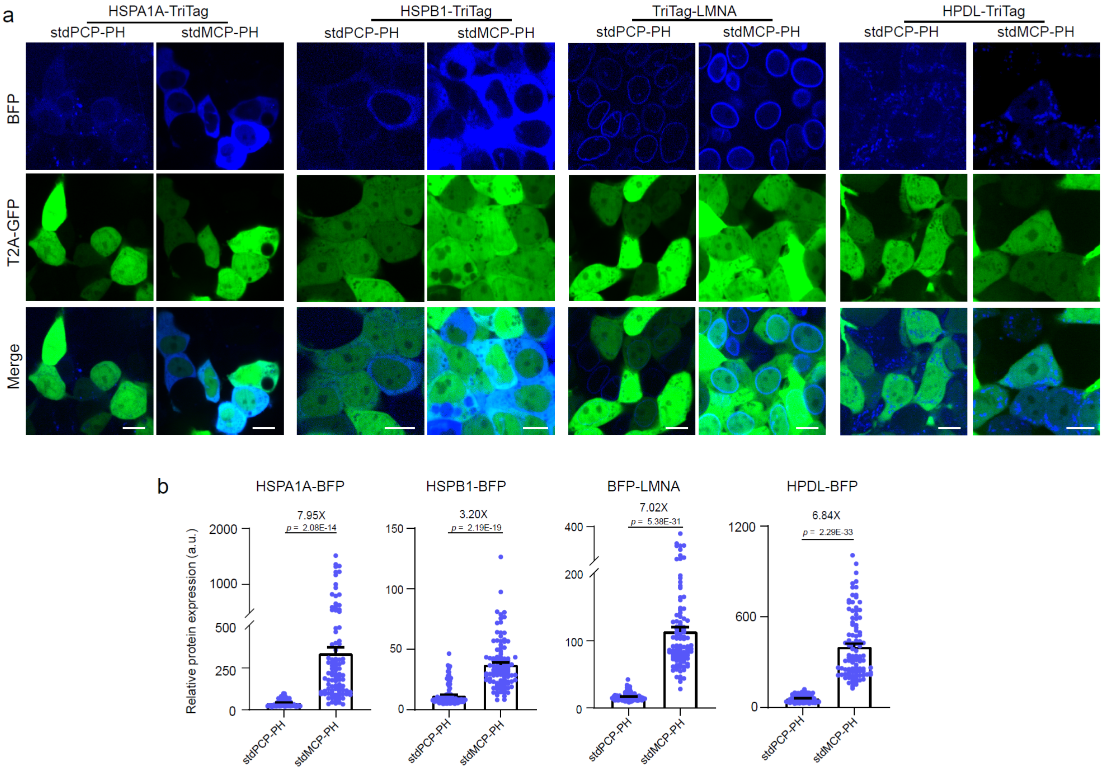

Supplementary Fig. 6 Activation of endogenous genes by Narta in 293T cells. a, Representative images of endogenous reporter genes labeled by BFP^{TriTag} with or without Narta activation. stdMCP-PH-T2A-GFP was transfected into cells to induce Narta activation, while stdPCP-PH-T2A-GFP was transfected to serve as the negative control. Scale bar, 5μm. **b**, Quantifications of protein expression levels based on fluorescent imaging in (**a**). Each dot represents a single cell. For each group from left to right, n = 94, 98, 103, 108, 100, 100, 102 and 102 cells, respectively. All plots are shown as mean ± s.e.m. *P*-value was analyzed by two-tailed Student's *t*-test. Source data are provided as a Source Data file.

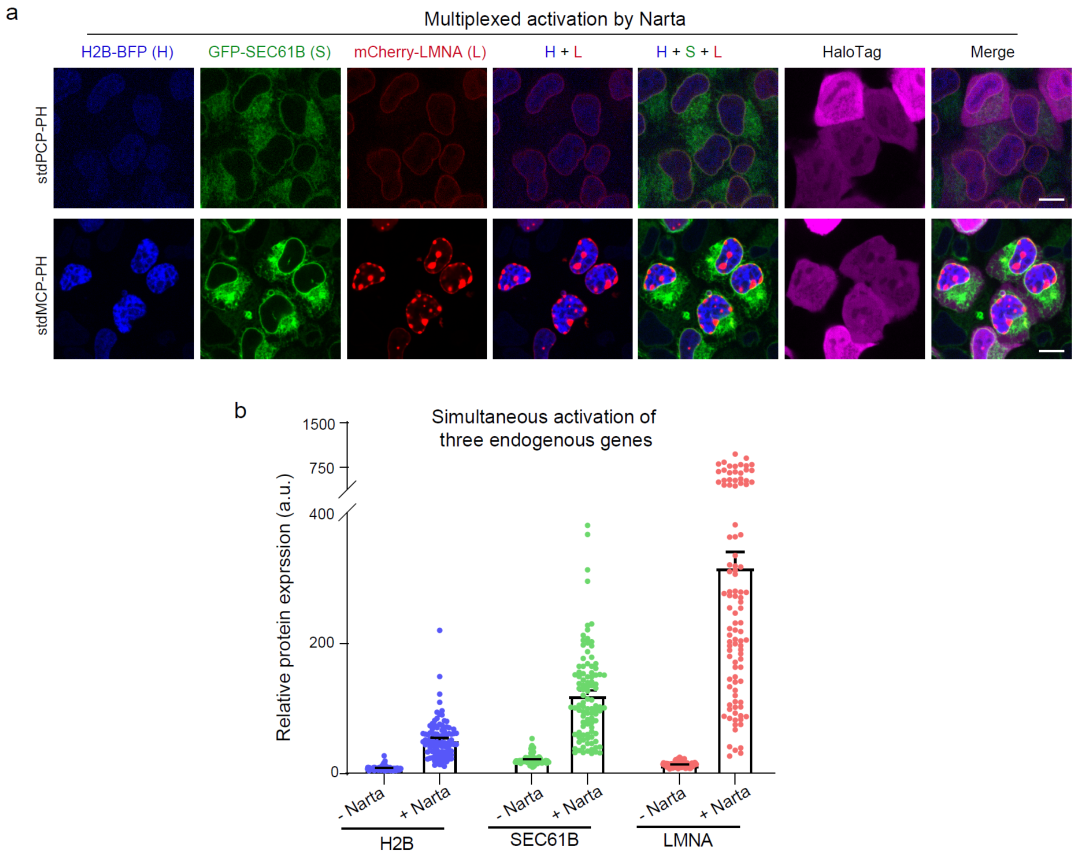

Supplementary Fig. 7 Simultaneous activation of three endogenous genes by Narta. a, A HeLa cell line harboring endogenous mCherry^{TriTag}-LMNA, H2B-BFP^{TriTag} and GFP^{TriTag}-SEC61B was transfected with stdMCP-PH-T2A-HaloTag to induce Narta multiplexed activation. stdPCP-PH-T2A-HaloTag was used as the negative control. **b**, Quantification of protein expression levels of the three target genes. Each dot represents a single cell (n = 100 cells). Data represent mean ±s.e.m. Source data are provided as a Source Data file.

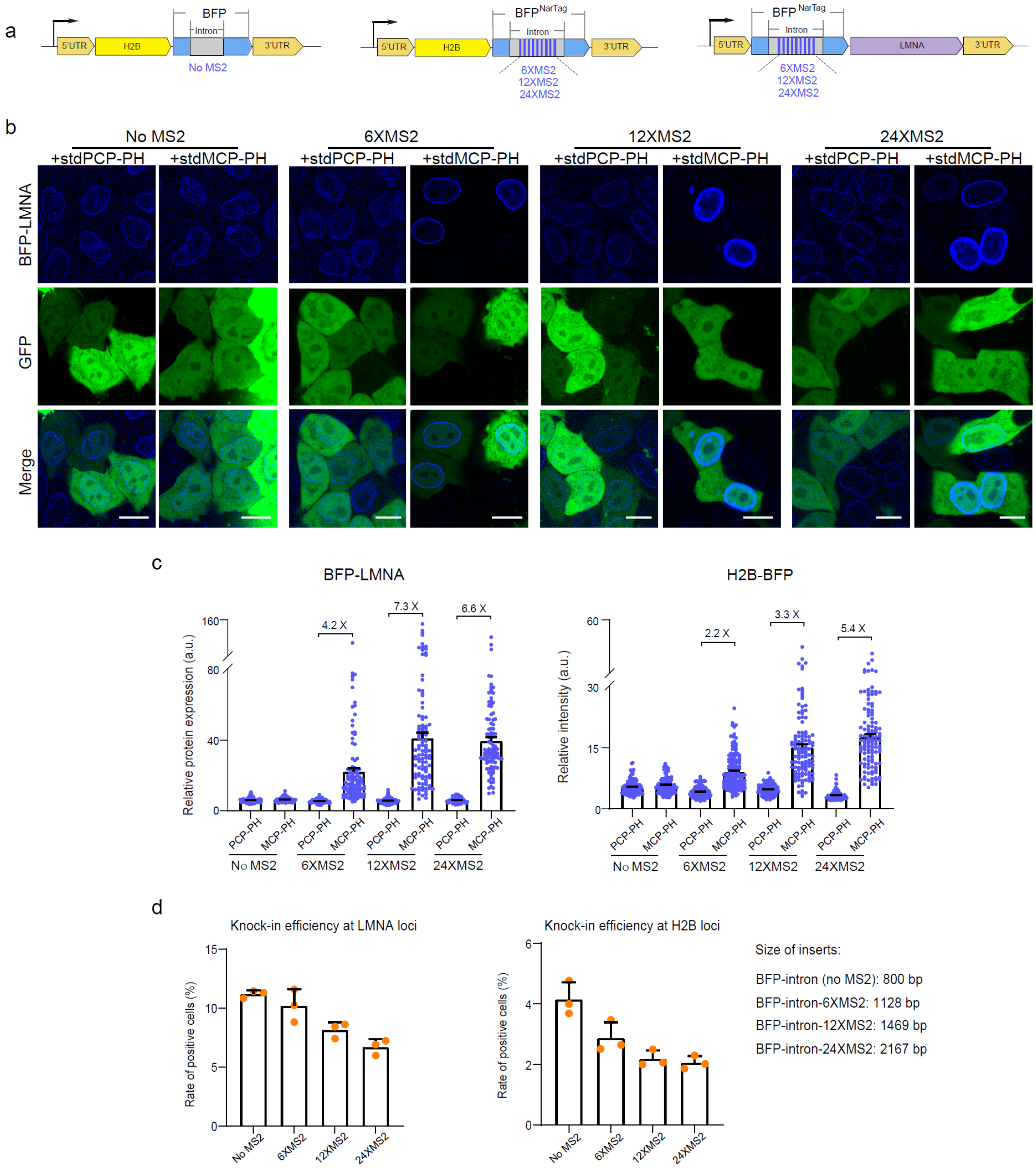

Supplementary Fig. 8 The optimal copy number of MS2 for Narta activation. a, Schematic depicting BFP^{NarTag} designs that harbor different copy numbers of MS2 repeats. NarTag was integrated into the C- or N-terminus of target genes by CRISPR editing. **b**, Representative images of endogenous reporters labeled by different BFP^{NarTags} with or without Narta activation. stdMCP-PH-T2A-GFP was transfected into cells to induce Narta activation, while stdPC-PH-T2A-GFP was used as the negative control. Scale bar, 5 µm. **c**, Quantifications of protein expression levels based on fluorescent imaging in (**a**). Each dot represents a single cell. n = 100 cells. Data represent mean ± s.e.m. **d**, Plots showing knockin rates of different NarTags at LMNA or H2B loci through homology-directed repair. NarTag knockin was quantified based on fluorescent imaging. Positive rate was calculated using the number of successfully edited cells (BFP$^+$) divided by the number of total cells containing CRISPR editing components (GFP$^+$). Data is shown as mean ± s.e.m. n = three biological replicates. Source data are provided as a Source Data file.

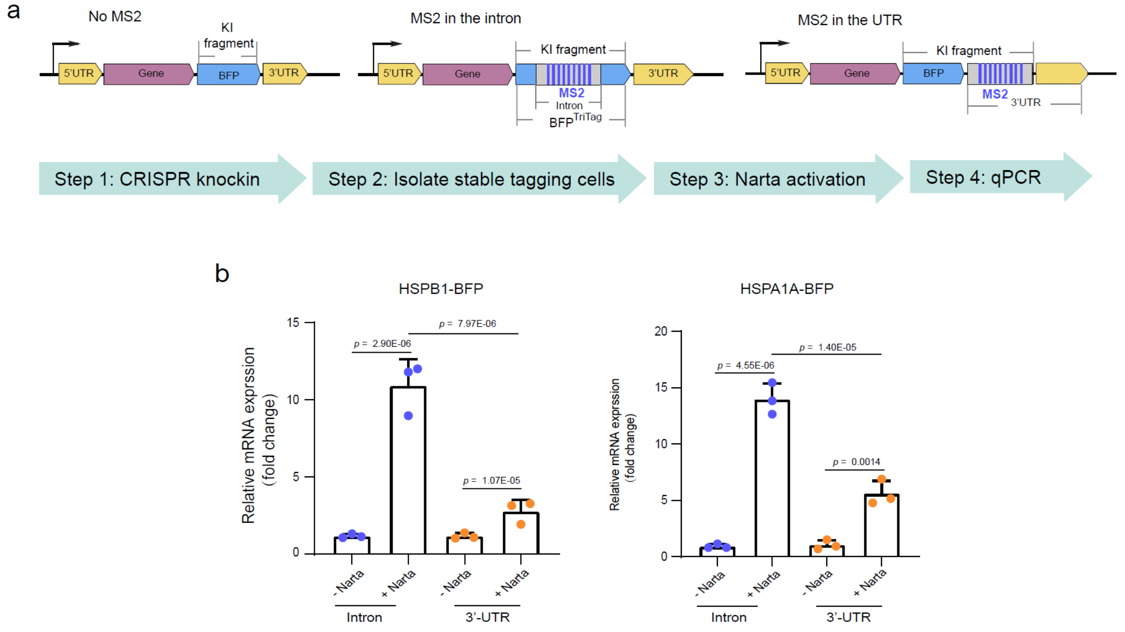

Supplementary Fig. 9 Narta activation via intronic MS2 is more effective than UTR MS2. a, Schematic diagram of two different MS2 positions (in the intron or UTR region) and the workflow to detect Narta activation by qRT-PCR. **b**, Quantitative analysis of mRNA abundance with or without Narta activation under different conditions by qRT-PCR. n = three biological replicates. Data is shown as mean ± s.e.m. *P*-value was determined by One-way ANOVA analysis (with Post Test, turkey). Source data are provided as a Source Data file.

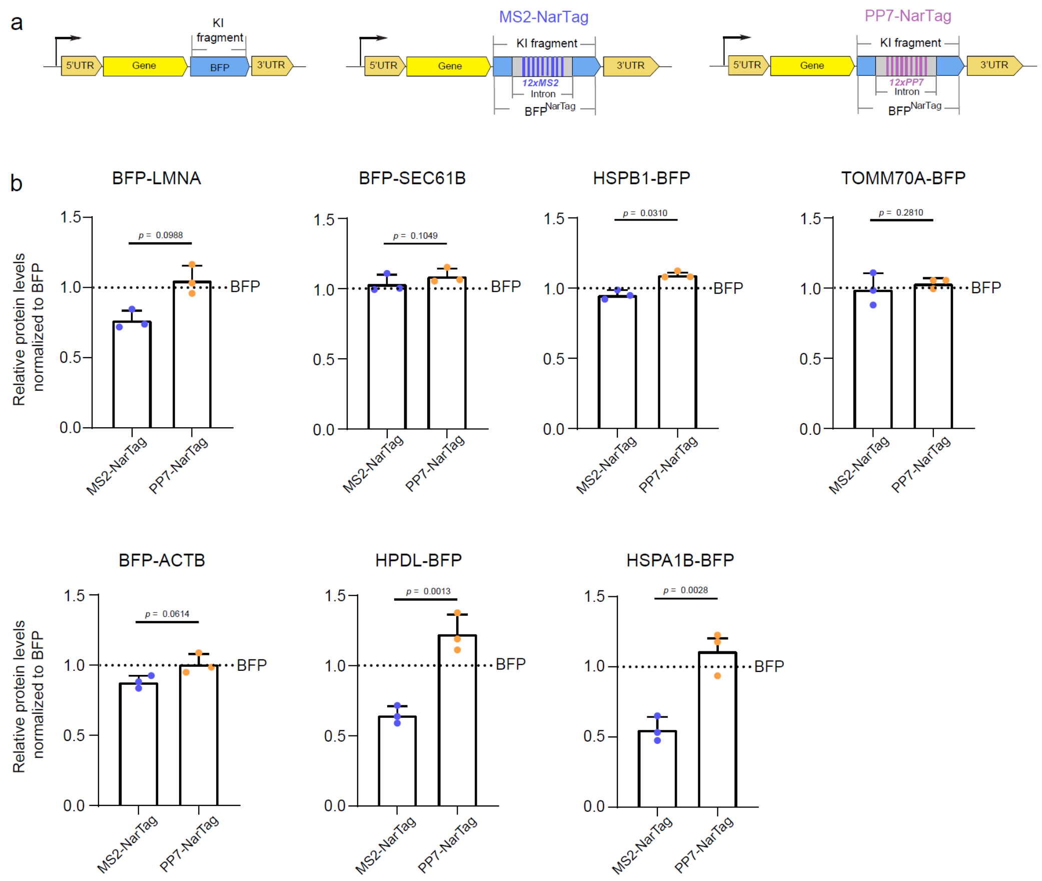

Supplementary Fig. 10 Effect of NarTag knockin on the protein expression of target genes. a, Schematic depicting different BFP^{NarTag} designs which harbor 12 copies of MS2 or PP7 repeats in the intron. **b**, Quantifications of protein abundance in BFP^{NarTag} knockin cells vs. BFP knockin cells by quantitative fluorescent imaging. n = 3 independent biological experiments. Fold changes were shown as mean ± s.e.m. *P*-value was analyzed by two-tailed Student's *t*-test. Source data are provided as a Source Data file.

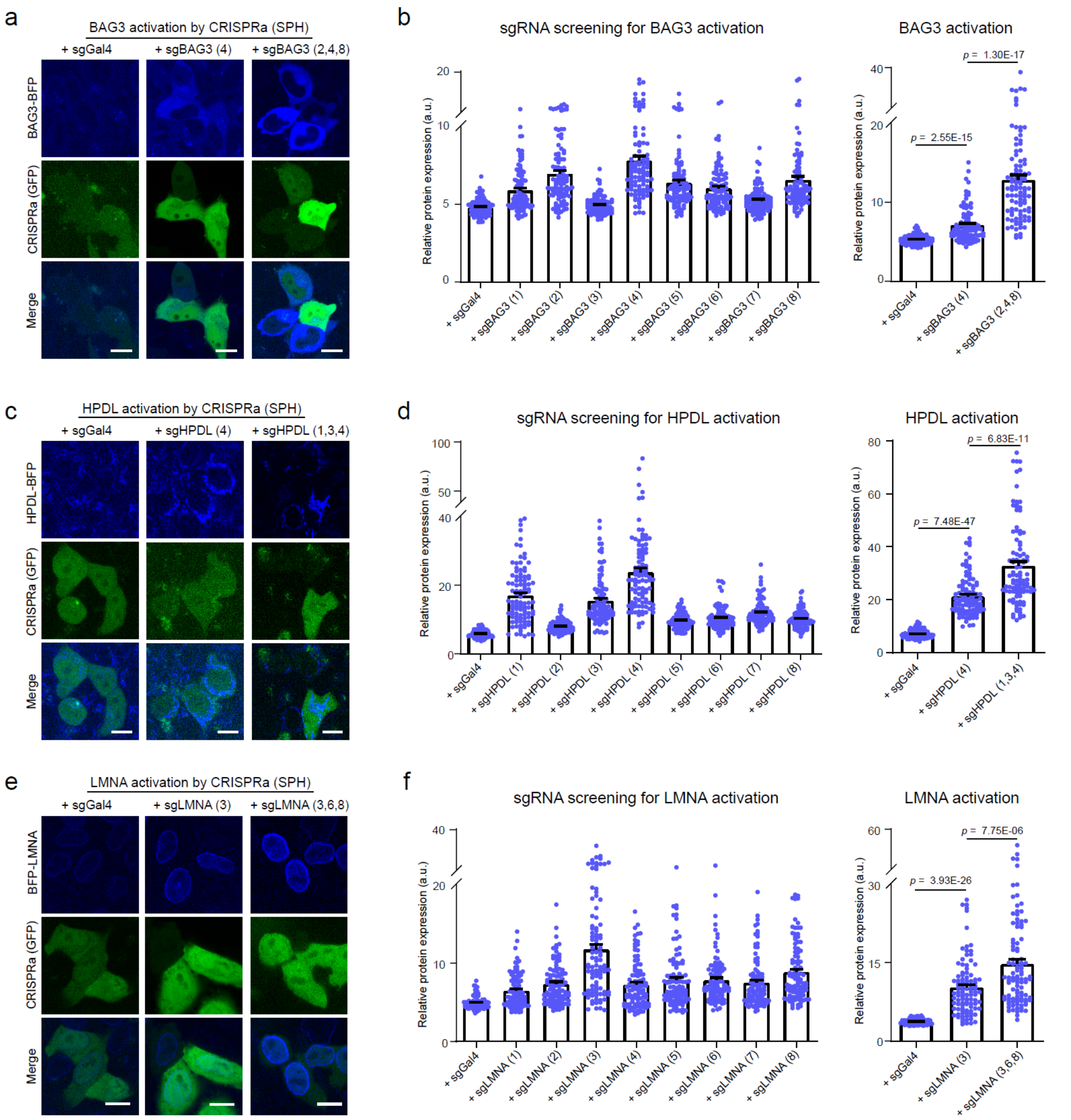

Supplementary Fig. 11 Selecting efficient sgRNAs to perform CRISPRa. **a**,**c**,**e**, Representative images showing CRISPRa-mediated endogenous gene activation, including BAG3-BFP, HPDL-BFP and BFP-LMNA. The negative control group used sgGal4 instead of sgRNAs of target genes. The reporter cell line was transfected with the indicated sgRNAs and dCas9-SPH activator (dCas9-10xGCN4 and scFV-p65-HSF1). Scale bar, 10 μm. **b**,**d**,**f**, Measurements of protein abundance of CRISPRa target genes by quantitative fluorescent imaging in HeLa cells in which the indicated individual or combinational use of sgRNAs were co-expressed with dCas9-SPH. Each dot represents a single cell. n = 100 cells. Data represent mean ± s.e.m. *P*-value was determined by One-way ANOVA with Turkey's post hoc test. Source data are provided as a Source Data file.

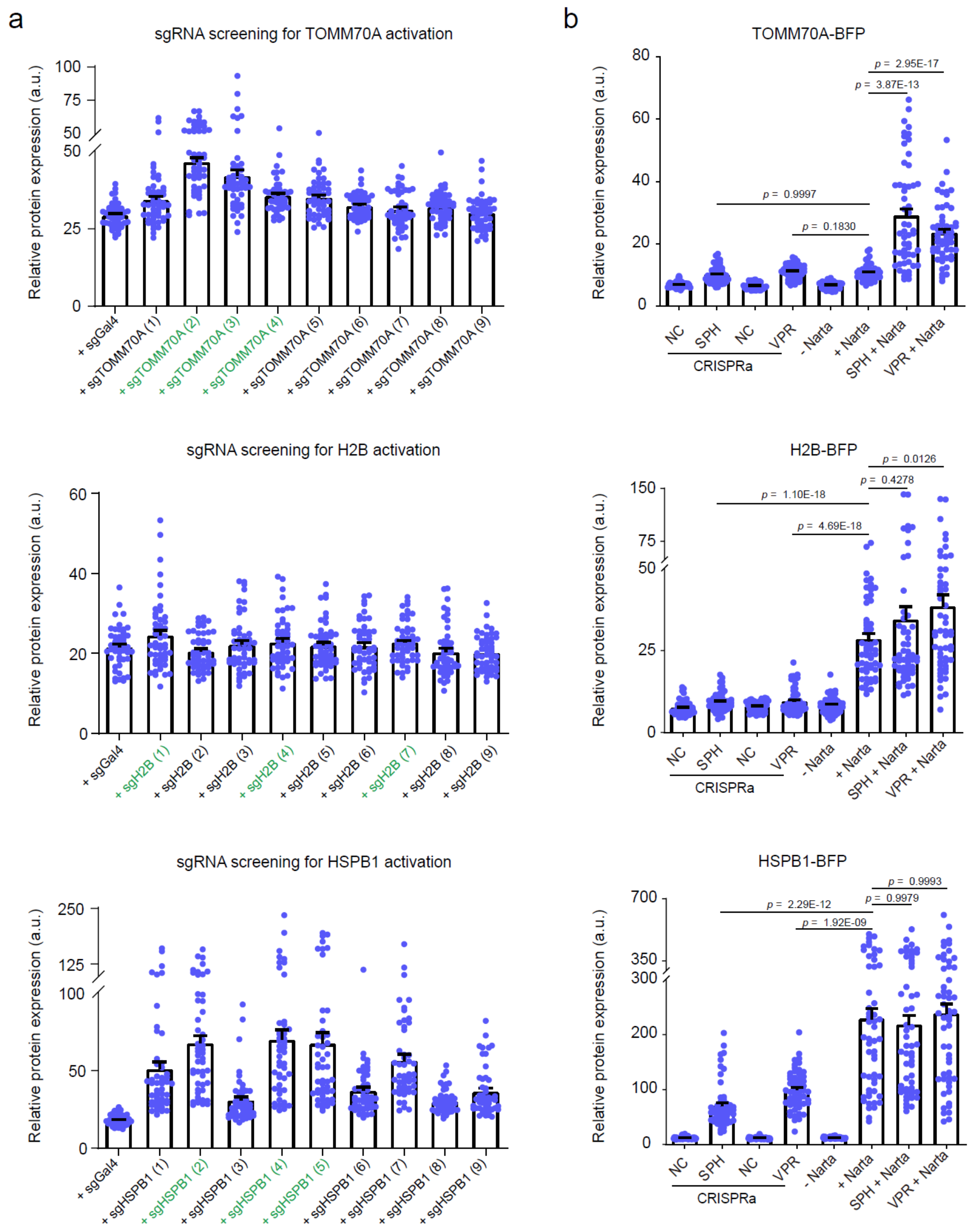

Supplementary Fig. 12 Comparison and combinatory use of CRISPRa and Narta for gene activation. a, sgRNA screening for CRISPRa. sgRNA targeting Gal4 was used as the negative control. Individual sgRNAs were tested to activate TOMM70A, H2B and HSPB1 which were tagged with BFP^{TriTag}. BFP fluorescent intensity was measured to indicate relative protein expression levels of target genes following CRISPRa activation by dCas9-SunTag-10xPH (SPH). For each target gene, nine sgRNAs were tested. Three sgRNAs that achieved relatively more efficient activation than others were labeled by green and selected for the use in (b). **b**, Quantifications of protein expression level based on fluorescent imaging under various conditions, including gene activation mediated by CRISPRa (two strategies, SPH and dCas9-VPR), Narta or combinatorial use of both. CRISPRa was performed by using the three selected sgRNAs (labeled by green) from (a). Each dot represents a single cell. n = 50 cells. Data is shown as mean ± s.e.m. *P*-value was determined by One-way ANOVA with Turkey's post hoc test. Source data are provided as a Source Data file.

a

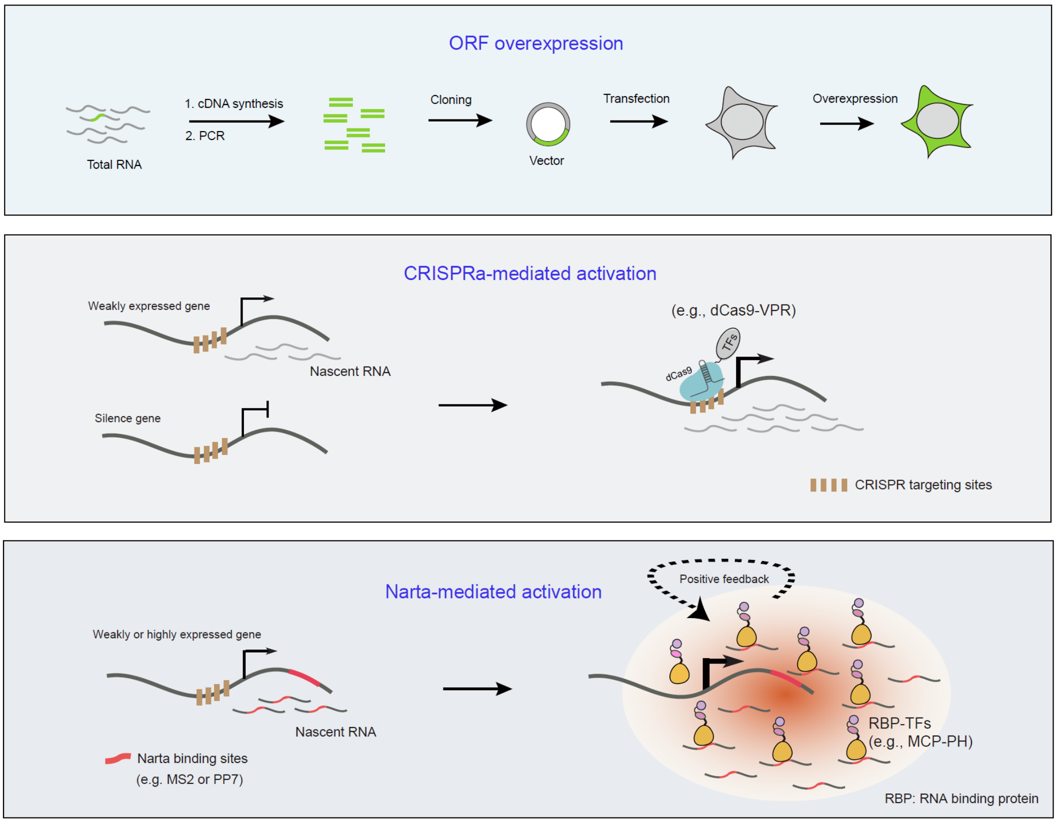

b

Activation method	Target gene	Throughput	Off-target effects	No. of components required	Advantages or limitations
ORF overexpression	Exogenous	Low	Nonexistent	1	Increase expression with a wide dynamic range<br>GC-rich or long genes may be difficult to clone
CRISPRa	Endogenous	High	Minimal	2 or 3 (sgRNA, activation modules)	Applied for genome-wide screen<br>1) Limited targeting sites<br>2) Uncertainty of sgRNA efficacy<br>3) Difficult to activate highly-expressed genes
Narta	Exogenous & Endogenous	Low (current stage)	Minimal	1	Activate genes with a wide dynamic range<br>1) Need to edit the genome (current stage)<br>2) May only activate expressed genes

Supplementary Fig. 13 Comparison of Narta to other gene activation methods. **a**, Schematic of three gene activation systems, including ORF overexpression, CRISPRa and Narta. dCas9-guided transcription factors bind to limited targeting sites around the transcription start site (TTS). Narta activates genes by recruiting abundant transcription factors to nascent RNAs. The higher transcriptional level induced by Narta will recruit more transcription factors that will further activate the target gene, displaying as positive feedback. **b**, Summary of advantages and limitations of the three gene activation systems.

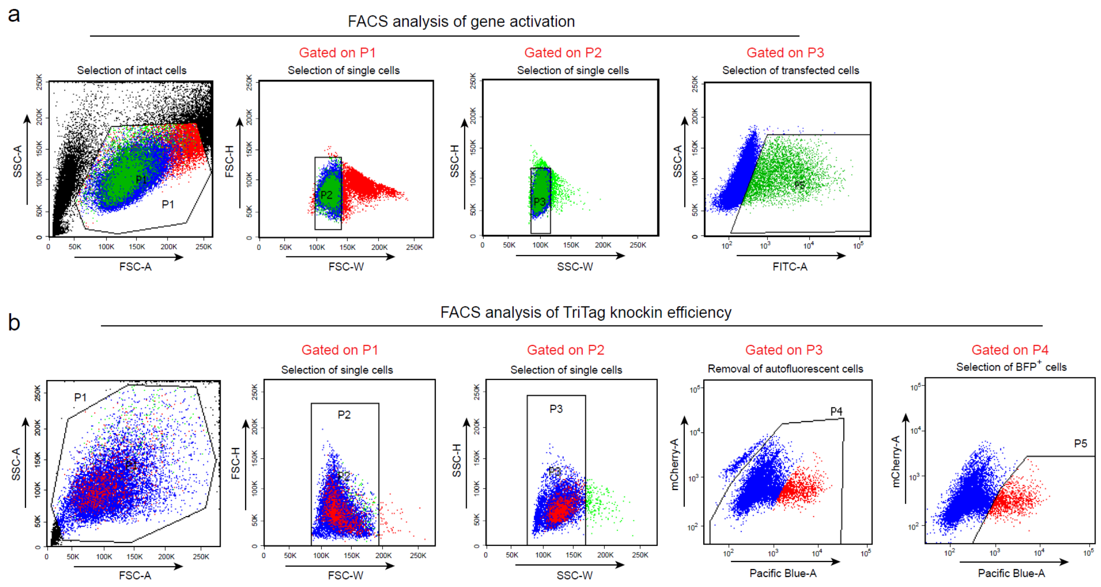

Supplementary Fig. 14 Gating strategies of flow cytometry analysis. **a**, Flow cytometry analysis of gene activation, refers to Fig 4c. **b**, Flow cytometry analysis of BFPTriTag knockin efficiency, refers to Fig 5a.

Supplementary Table 1. CRISPR-Cas9 sgRNAs used in this study

sgRNA name	Target sequence	PAM	Purpose
sgH2B	GCGAGCGCCAGGTCCCGGCA	GGG	TriTag knockin
sgLMNA	GCCATGGAGACCCCGTCCCAG	CGG	TriTag knockin
sgHSPA1A	CATTGAGGAGGTAGATTAG	GGG	TriTag knockin
sgHSPA1B	CATTGAGGAGGTGGATTAG	GGG	TriTag knockin
sgHSPB1	CGCCAAGTAAAGCCTTAGCC	CGG	TriTag knockin
sgHSPB8	GAGCAGCCACCATGGCTGA	CGG	TriTag knockin
sgHPDL	GGAAGCCTAAGGATGCCCA	GGG	TriTag knockin
sgBAG3	GTCTGATTTTTACAGGGCAG	AGG	TriTag knockin
sgMAP4	GCAGTTGCAGTGGTGCAGAA	TGG	TriTag knockin
sgCTCF	GCACAAGGCTCCGCCATCAC	CGG	TriTag knockin
sgCYB5B	AATCTCAGTTAGCGGTGGAG	AGG	TriTag knockin
sgACTB	GCCGTTGTCGACGACGAGCG	CGG	TriTag knockin
sgNUP35	CTGGTAGTAGAACACCAAGA	AGG	TriTag knockin
sgVAPB	GCCGCTAAGGAACATGGCGA	AGG	TriTag knockin
sgCBX1	GAAAGCTGGCGGGCACTATG	GGG	TriTag knockin
sgCLTA	AGCCATGGCGGGCAACTGAA	CGG	TriTag knockin
sgSEC61B	GCTTGTCTCCCTCTACAGCC	TGG	TriTag knockin
sgTOMM70A	CCACCAACATTATAAAACAG	GGG	TriTag knockin
sgP300	ATCTCCGAAAGAATTAAAAA	TGG	HaloTag knockin
sgBRD4	TGGGATCACTAGCATGTCTG	CGG	HaloTag knockin
sgTS1	GCACCGATGCTCTCCGAGG	AGG	Design of TriTag
sgTS2	GGAGCTTACTGAGACTCTTC	GGG	Design of double cut donor
sgminiCMV	GTCCCCTCCACCCCACAGTG	GGG	CRISPRa
sgBAG3-1	CGATTATAGCCGATGACTCA	GGG	CRISPRa
sgBAG3-2	GTCATCGGCTATAATCGCGG	CGG	CRISPRa
sgBAG3-3	GCACGCGCACGAGTCGGAAG	GGG	CRISPRa
sgBAG3-4	TCCGGCTTCCCACCCGCAGG	CGG	CRISPRa
sgBAG3-5	AAGCCGGACGAGCTGGACTG	GGG	CRISPRa
sgBAG3-6	ATGAAAGCCGAAACCCGCCC	GGG	CRISPRa
sgBAG3-7	GGAACACTCACTCGGCGCAA	AGG	CRISPRa
sgBAG3-8	GCGCCGAGTGAGTGTTCCCG	GGG	CRISPRa
sgHPDL-1	GCGCGAGCACAGGCACCGCG	TGG	CRISPRa
sgHPDL-2	GCCAAGCAGGAGAAAGAGGG	CGG	CRISPRa
sgHPDL-3	GTATCCGAGGCGACAGGGTG	TGG	CRISPRa
sgHPDL-4	GGCCCGGGGAAAGAGCAGCG	CGG	CRISPRa
sgHPDL-5	CAAGGATGTGACCAGGCGTG	AGG	CRISPRa
sgHPDL-6	AGGCAGCCATTGTGAGTTCC	TGG	CRISPRa
sgHPDL-7	CCAACTCTTTCCCTGAACCC	GGG	CRISPRa
sgHPDL-8	CCCTTCGCTGTGAGGTTGGG	GGG	CRISPRa
sgLMNA-1	GAGGGGCCACTACTCTTAA	AGG	CRISPRa
sgLMNA-2	GAAGGACCGTTGGGGTGTGG	CGG	CRISPRa
sgLMNA-3	GAAGGACCGTTGGGGTGTGG	GGG	CRISPRa

sgLMNA-4	AGCGCACCCCTAACTGCGTG	AGG	CRISPRa
sgLMNA-5	AGTTAGGGGTGCGCTGGAGA	GGG	CRISPRa
sgLMNA-6	CCAGAAGGTCTGAGGCAATG	GGG	CRISPRa
sgLMNA-7	GGGGAGAAAGAGAGGGAAGT	GGG	CRISPRa
sgLMNA-8	CTAGAGGGCAGAGCCAAGGA	GGG	CRISPRa
sgH2B-1	AAGGTCCGCACACGGACCAA	TGG	CRISPRa
sgH2B-2	CAATGGAAATGAACGACTTT	CGG	CRISPRa
sgH2B-3	GAGCCCTAATTTGCATAAGG	TGG	CRISPRa
sgH2B-4	CGGCCAATCAAGTCAAGAGT	CGG	CRISPRa
sgH2B-5	TCCTAATATGGCTTCAGAAC	CGG	CRISPRa
sgH2B-6	GTCAAGAACGAGTTTTTCAT	TGG	CRISPRa
sgH2B-7	AAGATTGAAAGAGAACCAAG	AGG	CRISPRa
sgH2B-8	GCCTTTATAAGCTTTCCTAG	GGG	CRISPRa
sgH2B-9	TTCAATCTTATTTTGTTGCG	AGG	CRISPRa
sgHSPB1-1	GCGTTCAGGGGCCGTGGGCG	GGG	CRISPRa
sgHSPB1-2	CGAGAGAAGGTTCCAGATGA	GGG	CRISPRa
sgHSPB1-3	AAGAGGGTTCAGCCCTCATC	TGG	CRISPRa
sgHSPB1-4	CCAGCAACGCTTAAGCACCA	GGG	CRISPRa
sgHSPB1-5	GCGACGCTGGAGCCGTGCCA	CGG	CRISPRa
sgHSPB1-6	GCTGGAGCCGTGCCACGGCA	GGG	CRISPRa
sgHSPB1-7	GCTCTGTCCTCCTTAACAGA	AGG	CRISPRa
sgHSPB1-8	ATACAACTGAGAAGTGGCAG	AGG	CRISPRa
sgHSPB1-9	GAGCCAGACAGGTTGGGTTG	GGG	CRISPRa
sgTOMM70A-1	CGTGGCTATAACTGGAAAAC	TGG	CRISPRa
sgTOMM70A-2	AGGTTCAAGAGGTGCCAACG	TGG	CRISPRa
sgTOMM70A-3	AACCGTGCTAACGGCCACAG	AGG	CRISPRa
sgTOMM70A-4	CGAGAAACCAACGGATTAGA	AGG	CRISPRa
sgTOMM70A-5	AAAGAGCTCACCGCTGAAGA	AGG	CRISPRa
sgTOMM70A-6	GATGCAGCAGCCTTCTTCAG	CGG	CRISPRa
sgTOMM70A-7	AACATAGCGAGATTTTAGTT	TGG	CRISPRa
sgTOMM70A-8	GGTTATAGGGGAGATAGCGA	TGG	CRISPRa
sgTOMM70A-9	TAGGTGGAAATGGCACATCT	AGG	CRISPRa

Note: If the first nucleotide of target sequence is not “G”, a “G” would replace it for better transcription under U6 promoter.

Supplementary Table 2. CRISPR-Cas13 crRNAs used in this study

crRNA name	Target sequence	Purpose
crNC	TCACCAGAAGCGTACCATACTCACGAACAG	Negative control
crTS1	CCTCCTCGGAGAGCATCGGTGC	Targeting TriTag

Supplementary Table 3. Primers used for qPCR

Gene of interest	5’-oligo	3’-oligo
UBC	CCCAGTATCAGCAGAAGGACA	ATCGCCGAGAAGGGACTACTT
BFP^{TriTag}	GAGAACATGCACATGAAGCTG	GAAGCTAGTAGCCAGGATGTCG
