# Supplementary material for: Gene activation guided by nascent RNA-bound transcription factors
Source: Nat Commun. 2022 Nov 28;13:7329. doi: 10.1038/s41467-022-35041-7 (PMC9705438; doi:10.1038/s41467-022-35041-7)
Supplement: Supplementary file 3 — Description of additional Supplementary File [file 41467_2022_35041_MOESM3_ESM.pdf]

Descriptions of additional Supplementary Files

Supplementary Video 1: Transcriptional dynamics of miniCMV-BFPTriTag under normal condition. Real-time imaging was performed to monitor the transcriptional bursting of miniCMV-BFPTriTag without adding Dox to induce Narta activation. The production of nascent RNAs was revealed by stdMCPtdTomato (red). 2 h movie is shown. 11 z-plane images spaced by 0.3 µm were acquired every 2 min using a spinningdisk confocal microscope. Scale bar, 5 µm.

Supplementary Video 2: Transcriptional dynamics of miniCMV-BFPTriTag upon Narta activation. In the Doxycycline-inducible Narta system, the addition of Dox resulted in the expression of stdMCP-T2A-GFP, leading to transcriptional activation of miniCMVBFPTriTag. The accumulation of nascent RNAs were indicated by stdMCPtdTomato (red). 2 h movie is shown. 11 z-plane images spaced by 0.3 µm were acquired every 2 min using a spinningdisk confocal microscope. Scale bar, 5 µm.

Supplementary Video 3: Transcriptional bursting of H2B-BFPTriTag under control condition. The endogenous H2B was tagged with BFPTriTag at its C-terminus. stdMCPtdTomato (red) was expressed to label nascent RNAs produced at H2B loci. 12 h before confocal imaging, stdPCP-PHT2A-GFP was transfected to serve as a negative control for Narta activation. 2 h movie is shown. 11 z-plane images spaced by 0.3 µm were acquired every 2 min using a spinning-disk confocal microscope. Scale bar, 5 µm.

Supplementary Video 4 : Transcriptional bursting of H2B-BFPTriTag upon Narta activation. The endogenous H2B was tagged with BFPTriTag at its C-terminus. The nascent transcripts of H2B loci were labeled stdMCP-tdTomato (red). 12 h before confocal imaging, stdMCP-PH-T2AGFP was transfected to induce Narta activation. 2 h movie is shown. 11 zplane images spaced by 0.3 µm Images were acquired every 2 min using a spinning-disk confocal microscope. Scale bar, 5 µm.
