# Supplementary material for: Gene activation guided by nascent RNA-bound transcription factors
Source: Nat Commun. 2022 Nov 28;13:7329. doi: 10.1038/s41467-022-35041-7 (PMC9705438; doi:10.1038/s41467-022-35041-7)
Supplement: Supplementary file 8 — Reporting Summary [file 41467_2022_35041_MOESM8_ESM.pdf]

nature research

Corresponding author(s): Wei Zou, Baohui Chen

Last updated by author(s): Nov 8, 2022

Reporting Summary

Nature Research wishes to improve the reproducibility of the work that we publish. This form provides structure for consistency and transparency in reporting. For further information on Nature Research policies, see our Editorial Policies and the Editorial Policy Checklist.

Statistics

For all statistical analyses, confirm that the following items are present in the figure legend, table legend, main text, or Methods section.

n/a	Confirmed	
☐	☒	The exact sample size (*n*) for each experimental group/condition, given as a discrete number and unit of measurement
☐	☒	A statement on whether measurements were taken from distinct samples or whether the same sample was measured repeatedly
☐	☒	The statistical test(s) used AND whether they are one- or two-sided<br>*Only common tests should be described solely by name; describe more complex techniques in the Methods section.*
☒	☐	A description of all covariates tested
☒	☐	A description of any assumptions or corrections, such as tests of normality and adjustment for multiple comparisons
☐	☒	A full description of the statistical parameters including central tendency (e.g. means) or other basic estimates (e.g. regression coefficient) AND variation (e.g. standard deviation) or associated estimates of uncertainty (e.g. confidence intervals)
☐	☒	For null hypothesis testing, the test statistic (e.g. *F*, *t*, *r*) with confidence intervals, effect sizes, degrees of freedom and *P* value noted<br>*Give P values as exact values whenever suitable.*
☒	☐	For Bayesian analysis, information on the choice of priors and Markov chain Monte Carlo settings
☒	☐	For hierarchical and complex designs, identification of the appropriate level for tests and full reporting of outcomes
☐	☒	Estimates of effect sizes (e.g. Cohen's *d*, Pearson's *r*), indicating how they were calculated

Our web collection on statistics for biologists contains articles on many of the points above.

Software and code

Policy information about availability of computer code

Data collection	Wide field fluorescent images were acquired by NIS-Elements on a Nikon Ti2-E fluorescence microscope. Confocal images were captured by CellSens on an Olympus IX83 fluorescence microscope equipped with spinning-disk confocal scanner. Super-resolution images were performed using commercialized Hessian-SIM termed HIS-SIM (High Intelligent and Sensitive Microscope) provided by Guang zhou Computational Super-resolution Biotech Co., Ltd. FACS data was collected by MoFlo Astrios EQ (Beckman) and BD Fortessa instrument (BD Biosciences).
Data analysis	ImageJ software was used to analyze fluorescence imaging data for calculating the mean intensity of fluorescent reporter proteins and the total intensity of fluorescent spots (representing nascent RNA, MED1, p300 or BRD4 signals). Line scan was obtained using the 'Analyze/Plot Profile' function (a plugin for ImageJ). The extracted parameters were then analyzed in Excel and plotted using GraphPad Prism. GraphPad Prism (Version 8, GraphPad Software, La Jolla, CA, USA, https://www.graphpad.com) was used to calculate the mean values, the standard error of the mean (SEM) and correlation coefficient (r) for the statistical analysis. The statistical significance between two groups was calculated via student t-test, and significance among three or more groups was calculated using one-way ANOVA. FACS data was analyzed using FlowJo v10 software (FlowJo LLC). To analyze RNA-Seq data, normalized gene expression of each gene was obtained by function count from DESeq2 package (https://github.com/mikelove/DESeq2). The edgeR package (https://bioconductor.org/packages/release/bioc/html/edgeR.html) was then used to perform differential expression analyses between control and Narta with default parameters. The differential expression genes (up-regulated) were defined by a Benjamini-Hochberg adjusted p value.

For manuscripts utilizing custom algorithms or software that are central to the research but not yet described in published literature, software must be made available to editors and reviewers. We strongly encourage code deposition in a community repository (e.g. GitHub). See the Nature Research guidelines for submitting code & software for further information.

Data

Policy information about availability of data

All manuscripts must include a data availability statement. This statement should provide the following information, where applicable:

- Accession codes, unique identifiers, or web links for publicly available datasets
- A list of figures that have associated raw data
- A description of any restrictions on data availability

Raw-data of RNA-Seq has been deposited in the Gene Expression Omnibus (GEO) database under the accession number GSE204666. Source data are provided with this paper. Key plasmids will be deposited to Addgene.

Field-specific reporting

Please select the one below that is the best fit for your research. If you are not sure, read the appropriate sections before making your selection.

☒ Life sciences ☐ Behavioural & social sciences ☐ Ecological, evolutionary & environmental sciences

For a reference copy of the document with all sections, see nature.com/documents/nr-reporting-summary-flat.pdf

Life sciences study design

All studies must disclose on these points even when the disclosure is negative.

Sample size	Sample size were determined based on extensive preliminary experiments and previous similar studies. The sample size of each experiment is provided in the figure legends in main manuscript and supplementary information files. These numbers were sufficient for conducting statistical analysis.
Data exclusions	No data were excluded from the analyses.
Replication	Most of assays were carried out several times. All results were reliably reproduced at least once.
Randomization	No allocation was performed in this study. All the cell samples observed were randomly selected.
Blinding	Blinding was not done because the data acquisition and analysis were conducted under identical criteria/conditions/parameters in each comparison. However, all key experiments had been repeated by the two students independently.

Reporting for specific materials, systems and methods

We require information from authors about some types of materials, experimental systems and methods used in many studies. Here, indicate whether each material, system or method listed is relevant to your study. If you are not sure if a list item applies to your research, read the appropriate section before selecting a response.

Materials & experimental systems

n/a	Involved in the study	
☐	☒	Antibodies
☐	☒	Eukaryotic cell lines
☒	☐	Palaeontology and archaeology
☒	☐	Animals and other organisms
☒	☐	Human research participants
☒	☐	Clinical data
☒	☐	Dual use research of concern

Methods

n/a	Involved in the study	
☒	☐	ChIP-seq
☐	☒	Flow cytometry
☒	☐	MRI-based neuroimaging

Antibodies

Antibodies used	anti-GFP antibody (EarthOx, E022280, dilution: 2000x); anti-β-Actin Rabbit mAb (Sangon Biotech, D191047, dilution: 5000x); anti-MED1 Antibody, Affinity Purified (A300-793A, Bethyl, dilution: 1000x); Donkey Anti-Rabbit IgG H&L conjugated with Alexa Fluor® 647) (Abcam, ab150075, dilution: 1000x)
Validation	Validation statements are available from manufacturers:<br>GFP, https://earthox.net/product/anti-gfp-tag-rabbit-polyclonal-antibody-e022200-03/<br>Actin, https://www.sangon.com/productDetail?productInfo.code=D191047<br>MED1, https://www.citeab.com/antibodies/655647-a300-793a-rabbit-anti-med1-antibody-affinity-purifie<br>Donkey Anti-Rabbit IgG, https://www.abcam.com/Donkey-Rabbit-IgG-HL-Alexa-Fluor-647-ab150075.html

Eukaryotic cell lines

Policy information about cell lines

Cell line source(s)	HEK293T and HeLa cell lines were obtained from the ATCC. CHO-K1 was purchased from national collection of authenticated cell cultures (https://www.cellbank.org.cn/search-detail.php?id=536).
Authentication	Cells were authenticated by the vendor, no other authentications were conducted.
Mycoplasma contamination	All cell lines used were tested negative for Mycoplasma contamination.
Commonly misidentified lines (See ICLAC register)	No commonly misidentified cell lines were used.

Flow Cytometry

Plots

Confirm that:

☒ The axis labels state the marker and fluorochrome used (e.g. CD4-FITC).

☒ The axis scales are clearly visible. Include numbers along axes only for bottom left plot of group (a 'group' is an analysis of identical markers).

☐ All plots are contour plots with outliers or pseudocolor plots.

☒ A numerical value for number of cells or percentage (with statistics) is provided.

Methodology

Sample preparation	All experiments were done on well-established cell culture lines. Cells were collected and washed with PBS, and lifted by enzymatic treatment (trypsin) and finally suspended in PBS.
Instrument	BD Fortessa instrument (BD Biosciences) for FACS analysis in Fig 4b and Fig 5a; MoFlo Astrios EQ (Beckman) for the selection of knockin positive cells.
Software	FlowJo_V10
Cell population abundance	To improve knock-in efficiency by Narta, CRISPR-mediated TriTag knock-in was performed with additional supplement of 300ng stdMCP-PH-T2A-GFP (Narta activation) or tdPCP-PH-T2A-GFP (negative control) plasmids. Positive cells were gated using BFP as reporters. As many cells as possible were isolated for validating NarTag knockin. To compare the activation efficiency of CRISPRa, Narta and co-activation of CRISPRa and Narta, TriTag (BFP) cell lines were plated onto 24-well plates and transfected with corresponding plasmids the next day. The cells positive for CRISPRa and Narta expression were gated using GFP or HaloTag as reporters.
Gating strategy	For FACS selection on MoFlo Astrios EQ (Beckman), cells were first gated for the intact cells by FSC/SSC plot and then gated for single cells based on FSC-A/FSC-H and SSC-A/SSC-H. Positive cells were sorted out via BFP, GFP and HaloTag for different purposes. For FACS analysis on BD Foretssa, cells were first gated for the intact cells by FSC/SSC plot and then gated for single cells based on FSC-A/FSC-W and SSC-A/SSC-W. Gating strategies for Fig 4b and Fig 5a were provided in Supplementary Fig. 14.

☒ Tick this box to confirm that a figure exemplifying the gating strategy is provided in the Supplementary Information.
