# Supplementary figures and images for: Gene activation guided by nascent RNA-bound transcription factors
Source: Nat Commun. 2022 Nov 28;13:7329. doi: 10.1038/s41467-022-35041-7 (PMC9705438; doi:10.1038/s41467-022-35041-7)

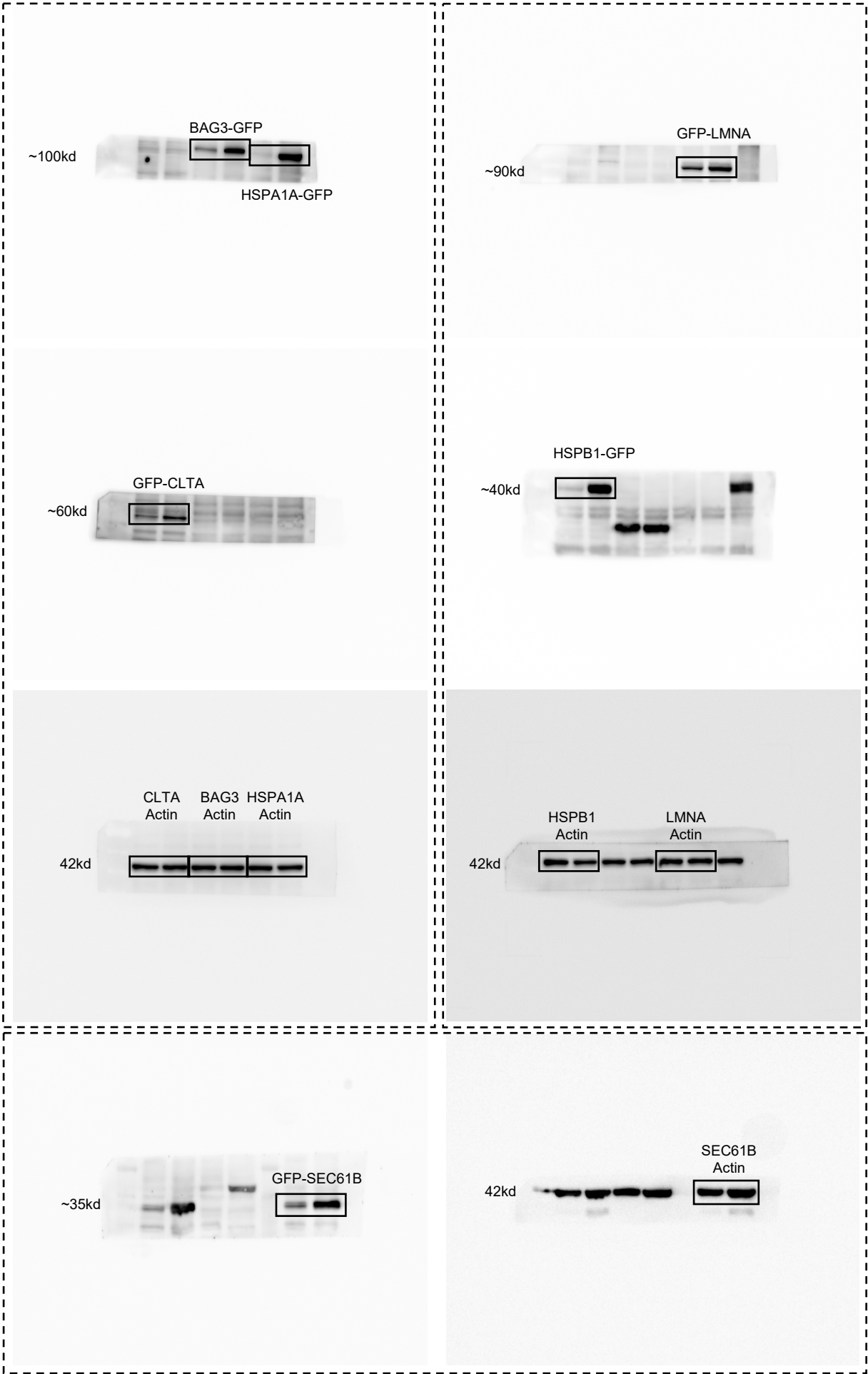

Supplement: Supplementary file 9 — Source Data [file 41467_2022_35041_MOESM9_ESM.zip › Source data files/Souce Data Figure 1.pdf]
